# Complexity of Fracturing in Terms of Non-Extensive Statistical Physics: From Earthquake Faults to Arctic Sea Ice Fracturing

**DOI:** 10.3390/e22111194

**Published:** 2020-10-22

**Authors:** Filippos Vallianatos, Georgios Michas

**Affiliations:** 1 UNESCO Chair on Solid Earth Physics and Geohazards Risk Reduction, Institute of Physics of the Earth’s Interior and Geohazards, Hellenic Mediterranean University Research Center, Crete, GR 73133 Chania, Greece; gmichas@chania.teicrete.gr; 2 Department of Geophysics–Geothermics, Faculty of Geology and Geoenvironment, National and Kapodistrian University of Athens, 15784 Athens, Greece

**Keywords:** fracturing, earthquakes, faults, sea ice time series, complexity, non-extensive statistical physics, scaling, extreme events

## Abstract

Fracturing processes within solid Earth materials are inherently a complex phenomenon so that the underlying physics that control fracture initiation and evolution still remain elusive. However, universal scaling relations seem to apply to the collective properties of fracturing phenomena. In this article we present a statistical physics approach to fracturing based on the framework of non-extensive statistical physics (NESP). Fracturing phenomena typically present intermittency, multifractality, long-range correlations and extreme fluctuations, properties that motivate the NESP approach. Initially we provide a brief review of the NESP approach to fracturing and earthquakes and then we analyze stress and stress direction time series within Arctic sea ice. We show that such time series present large fluctuations and probability distributions with “fat” tails, which can exactly be described with the *q*-Gaussian distribution derived in the framework of NESP. Overall, NESP provide a consistent theoretical framework, based on the principle of entropy, for deriving the collective properties of fracturing phenomena and earthquakes.

## 1. Introduction

Stress increase within solid Earth materials and the buildup of a proportional amount of strain eventually culminates in the deformation and fracture of the material. The most striking example in nature are earthquakes that mainly originate from the deformation and subsequent rupture of the seismogenic crust due to stress built-up arising from plate tectonic motions. As stress increases, cracks and fractures start to appear in the solid Earth that may coalesce to form larger fractures and eventually fault networks and tectonic plate boundaries [1,2]. Fracturing processes within solid Earth materials is inherently a complex phenomenon that incorporates a wide range of spatial and temporal scales and dynamics that interact nonlinearly to produce even extreme-in-size events [3,4]. The dynamics that lead to such events are generally unobservable in nature, while the exact physics and the microscopic laws that govern friction and the fracture evolution still remain elusive, so that the definition of the exact physics and forecasting of upcoming events represents nonetheless an outstanding challenge for science.

Despite the extreme complexity that characterize rupture initiation and propagation in solids, the ensemble of many fractures may present simple phenomenology and scaling properties that seem universally valid. The most prominent is scale-invariance that is manifested in the size distributions of earthquakes and faults. Fault trace-lengths and fault displacements manifest power-law type distributions and multifractal geometries [5], while earthquakes occur on a fractal-like network of faults with frequency-size distributions that scale according to the Gutenberg-Richter (G-R) relation [6], which resembles a power-law relationship between the number of earthquakes and the fault rupture area [7]. In addition, the temporal evolution of seismicity is characterized by multifractality and correlations at all timescales [8,9,10], while the production rate of aftershocks that follow a mainshock generally decays as a power-law with time according to the modified Omori formula [11].

Such properties have motivated the consideration of statistical physics as a consistent tool for explaining the macroscopic behavior of fracturing phenomena [4,12,13]. By using the laws of probability theory and statistics, statistical physics aims to provide theoretical insights and predict the macroscopic properties of such complex systems. While the prediction of particles’ motion within an ice block or within the deforming blocks of earthquake faults is infeasible, the ensemble average of this motion that results in the macroscopic behavior of the solid can be explained by statistical physics [14]. From a quantitative perspective, simple systems depend exponentially on time, space, energy and other basic variables, whereas complex systems behave subexponentially and typically as power-laws, with fracturing being a prototypical and very important example of complexity [15]. The later has recently motivated the application of non-extensive statistical physics (NESP) to the phenomenology of various complex systems, including fracturing phenomena and earthquakes [16]. NESP, originally introduced by [17], generalizes the classic Boltzmann-Gibbs statistical physics and its main advantage is that it considers all-length scale correlations among the various possible microstates, leading to heavy-tailed distributions with power-law asymptotic behavior. The application of NESP to various complex systems during the last two decades and the consistency between the theory and observations, have demonstrated that NESP is a suitable framework for illuminating the macroscopic properties of such systems by defining a priori the various microscopic states and their interactions.

In the present work we present a brief review and new results regarding the application of NESP to fracturing processes and earthquakes. For analytical reviews the reader can refer to [18,19,20]. Initially we provide the theoretical framework of NESP as it applies to fracturing processes and then discuss its application to earthquake related phenomena. Then we present for the first time the application of this framework to stress timeseries taken from Arctic sea ice. Stresses induced by ice motion demonstrate (multi)fractal scaling properties, anti-persistent behavior and “fat” tailed probability distributions [21,22], properties that cannot be described by Gaussian statistics. Instead, we show that even in the phenomenological level (i.e., without defining any underlying model) NESP framework can adequately describe stress fluctuations in Arctic sea ice. Such findings provide further insights in how to model risk of large deformation events that present large ice motion induced stresses, which can impact any given place in the Arctic sea ice pack.

## 2. Fracturing Processes in Terms of Non-Extensive Statistical Physics

In 1988, Tsallis [17] introduced the nonadditive entropy *S_q_* as a generalization of the classic Boltzmann-Gibbs (BG) entropy *S*_BG_. Although BG statistical mechanics properly describes nature for a wide class of physical systems that present short-ranged microscopic interactions (e.g., Markovian processes) and/or strongly chaotic dynamics, there is a significant class of physical systems that violate some or all of these properties [16,23,24]. Such systems typically present long-range correlations, multifractal geometries, intermittency and/or substantial variations between the various possible states, properties that typically lead to power-law type distributions. In contrast to BG statistical mechanics, NESP that refers to the nonadditive entropy *S_q_* contemplates all-length scale correlations among the various microscopic components of a system emanating to subexponential and typically heavy-tailed distributions. Such properties, i.e., intermittency, (multi)fractal structures, long-range correlations and power-law type distributions, seem to conform well to the collective properties of fracturing processes and earthquakes, as we discussed in the introduction of this article.

In the following, we address the NESP theory for a continuous variable *X* that may express the size of an earthquake in terms of the seismic moment M_o_, the size of a fractured fragment or a fault, or even the inter-event times and distances between successive earthquakes. If *p* (*X*) is the probability distribution of *X*, normalized such that 0 ≤ *p* (*X*) ≤ 1, then the non-additive entropy *S_q_* is expressed as:

(1) $$S_{q}=k\frac{1-\int p^{q}\left(X\right)dX}{q-1}$$

where *k* can be any constant, such as Boltzmann’s constant, and *q* is an entropic index that signifies the non-extensivity of the system. Let us note that the introduction of *S_q_* was originally inspired by multifractal geometries [16]. The notation *q* for the entropic index that is related to *S_q_* was adapted from the index variable *q* that denotes the order of the fluctuation function in multifractal sets, although the two indexes are not the same [16]. In Equation (1), the entropic index *q* interposes a bias in the probabilities of the various configurations, such that for 0 < *p* (*X*) < 1, *p^q^* (*X*) > *p* (*X*) for *q* < 1 and *p^q^* (*X*) < *p* (*X*) for *q* > 1 [16].

Now, to obtain *p*(*X*) the previous expression (Equation (1)) is optimized subjected to given constraints, the first being the normalization condition of *p*(*X*),

(2) $$\int_{0}^{\infty }p\left(X\right)dX=1\;$$

while the second refers to the condition of the generalized expectation value (or *q*-mean value) *X_q_*,

(3) $$X_{q}={\langle X\rangle }_{q}=\overset{\infty }{\underset{0}{\int }}XP_{q}\left(X\right)dX$$

where *P_q_*(*X*) is the escort probability distribution $P_{q}\left(X\right)=\frac{p^{q}\left(X\right)}{\int_{0}^{\infty }p^{q}\left(X\right)dX}$ [16]. Using the standard Lagrange multiplier method and the variational principle to Equation (1) under the constraints of Equations (2) and (3), the following probability distribution function is obtained:

(4) $$p\left(X\right)=\frac{{\left[1-\left(1-q\right)\beta_{q}X\right]}^{1/\left(1-q\right)}}{Z_{q}}=\frac{\exp_{q}\left(-\beta_{q}X\right)}{Z_{q}}$$

where *Z_q_* is the generalized partition function,

(5) $$Z_{q}=\int_{0}^{x_{max}}\exp_{q}\left(-\beta_{q}X\right)dX$$

and exp*_q_* (*X*) is the *q*-exponential function (see [16] and references therein), defined as:

(6) $$\exp_{q}\left(X\right)=\;\{\begin{array}{c} {\left[1+\left(1-q\right)X\right]}^{1/\left(1-q\right)}\;\mathrm{for}\;1+\left(1-q\right)X\geq 0\\ 0\;\mathrm{for}\;1+\left(1-q\right)X\leq 0 \end{array}$$

For *q* > 1, the *q*-exponential function presents asymptotic power-law behavior according to $\sim X^{-1/\left(q-1\right)}$, while for *q* < 1 a cut-off appears in the tail of the distribution at $X_{c}=1/\left(1-q\right)\beta_{q}$ [25]. The inverse of the *q*-exponential function (for *X* > 0) is the *q*-logarithmic function:

(7) $$\ln_{q}X=\frac{X^{1-q}-1}{1-q}$$

The previous functions, i.e., the *q*-exponential and *q*-logarithmic, recover the ordinary exponential and logarithmic functions, respectively, in the limit of *q*→1.

The corresponding to Equation (4) cumulative distribution function *P* (*X*) can be obtained upon integration of the escort probability distribution *P_q_* (*X*) (see [16,19,25]):

(8) $$P\left(X\right)=\overset{\infty }{\underset{0}{\int }}P_{q}\left(X\right)dX=\exp_{q}\left(-\frac{X}{X_{0}}\right)$$

where *P_q_*(*X*) and exp*_q_*(*X*) have been defined previously.

Another frequent case is when we impose the mean value of the squared variable *X*^2^ in Equation (3), which in this case provides the *q*-mean value $X_{q}^{2}$. In this case, optimization of *S_q_* under the constraints of normalization (Equation (2)) and the *q*-mean value $X_{q}^{2}$ leads to:

(9) $$p\left(X\right)=\frac{1}{Z_{q}}{\left[1-\beta \left(1-q\right)X^{2}\right]}^{1/\left(1-q\right)}$$

where $\beta ={\left[\left(3-q\right)X_{q}^{2}\right]}^{-1}$. The latter equation is known as the *q*-Gaussian distribution [16]. In the limit of *q*→1, the *q*-Gaussian converges to the ordinary Gaussian distribution, while for *q* > 1 it decays asymptotically as power-law, $\sim {\left|X\right|}^{-2/\left(q-1\right)}$.

## 3. Applications to Fracturing: From Earthquake Faults to Sea Ice

The principles of NESP have been applied in a series of recent publications to the macroscopic properties of fracturing and earthquakes and other earthquake-related phenomena ([18,19,20] and references therein). In these works, it has been illustrated that NESP constitute a powerful tool for deriving the collective properties of fracturing processes from the first principles of statistical physics and the specification of the microscopic interactions within the studied system. In the following, we initially provide a brief review to the various applications in earthquake fracturing phenomena and then apply for the first time the NESP framework to sea ice stress timeseries.

### 3.1. Applications to Earthquake Fracturing

Fracturing in lithosphere deformation is exemplified in fault networks. Fault networks that are typically the sites of smaller to larger magnitude earthquakes, represent a complex scale-invariant system with irregular geometries and sizes that vary from few millimeters to tens or hundreds of kilometers [2]. Scale-invariance in fault networks is further supported by fractal geometries that have been used to describe the growth patterns of complex fault networks [5]. The NESP approach to fault-size distributions arose naturally to provide a general principle, based on the notions of statistical physics, for deriving the least biased distribution that best describe fault and fracture systems [26,27]. The NESP analysis in a series of publications indicated that fault trace-length distributions can well be approximated with the *q*-exponential distribution for *q*-values greater than one, supporting subadditivity in planetary lithosphere deformation [26,27,28,29]. Furthermore, the reported *q*-values in fault-length distributions seem to depend on the tectonic environment [29], the mechanical correlations between the fault network [28], or on the strain rates in active continental rifts [27].

**Figure 1:**
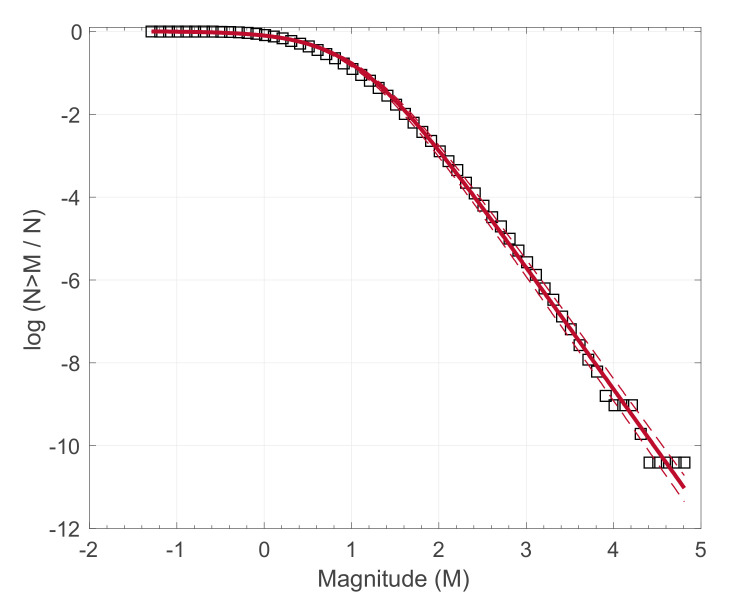
Frequency-magnitude distribution of earthquakes in the Yellowstone volcanic field (squares) during 1996–2016. The solid line represents the model of Equation (10) for *q_M_* = 1.44, while the dashed lines the corresponding 95% confidence intervals. Modified from [38].

Scale-invariant fracturing is further supported by the frequency-magnitude distribution of earthquakes that generally follow the Gutenberg–Richter relation [6], which resembles power-law scaling between the number of earthquakes and the fault rupture area (e.g., [7,18]). Consistent with the idea that earthquakes are primarily the result of stick-slip frictional instabilities inside fault zones, Sotolongo-Costa and Posadas [30], based on the NESP formalism, introduced the fragment-asperity interaction model for earthquake dynamics. According to this model, the released seismic energy *E* is related to the size of the fragments that fill the space between fault blocks. If *N* (>*M*) is the cumulative distribution of the number of earthquakes *N* with magnitude greater than *M*, then the derived model, as was later revised by [31,32], reads as:

(10) $$\frac{N\left(>M\right)}{N}={\left[\frac{1-\left(\frac{1-q_{M}}{2-q_{M}}\right)\left(\frac{10^{M}}{\alpha_{M}^{2/3}}\right)}{1-\left(\frac{1-q_{M}}{2-q_{M}}\right)\left(\frac{10^{M_{o}}}{\alpha_{M}^{2/3}}\right)}\right]}^{\left(2-q_{M}\right)/\left(1-q_{M}\right)}$$

where *M*_0_ is the minimum earthquake magnitude in the dataset, *a_M_* a model parameter that expresses the proportionality between the released seismic energy and the size of the fragments and *q_M_* the entropic index. The fragment-asperity model has found various applications in regional and local seismicity, as well as in volcanic seismicity [33,34,35,36,37,38]. In Figure 1 we show the application of the model to the 1996–2016 earthquake activity in the Yellowstone volcanic field (after the work of [38]). Generally, the results of the aforementioned studies suggest that the fragment-asperity model can adequately describe the frequency-magnitude distribution of earthquakes in a broader range of scales compared to the G-R relation. In addition, the *q_M_* temporal variations in regional seismicity have been used as an index of tectonic instability and proximity towards stronger earthquakes [39,40,41,42,43,44,45]. The combination of the *q_M_* temporal variations with natural time analysis of seismicity has shown precursory changes before strong earthquakes [41,45], including the 2011 Tohoku mega-earthquake [46]. Moreover, the combination of the aforementioned techniques reveals temporal correlations in the earthquake magnitudes evolution, which is further supported by the multifractal detrended fluctuation analysis of seismicity in the natural time domain [47].

**Figure 2:**
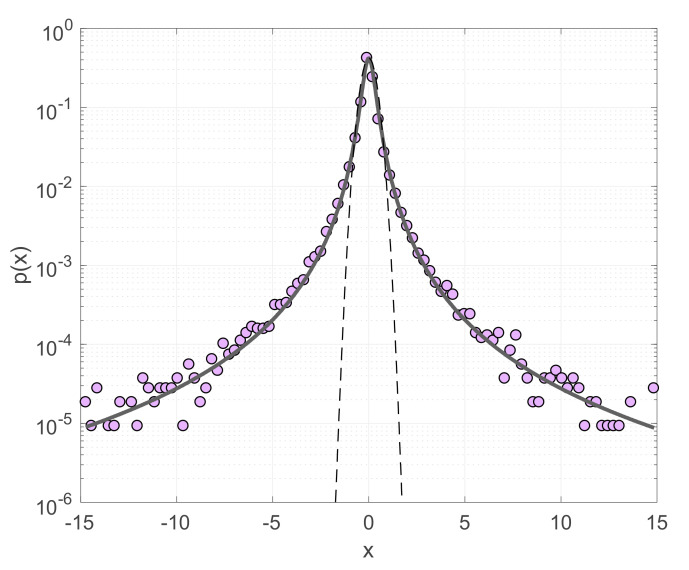
Probability density function *p* (*x*) of the normalized increments *x* (see text) of released seismic energies in the Southern California earthquake catalogue (filled circles) and the *q*-Gaussian fit (solid line) for *q* = 1.69. The Gaussian function (dashed line) is also shown for comparison.

Moreover, it has been shown that the probability distribution of incremental earthquake energies (i.e., the differences of released energies between successive earthquakes) presents heavy tails with asymptotic power-law scaling, a behavior that can well be reproduced by the *q*-Gaussian distribution (Equation (9)) [19,36,48]. In Figure 2 we show the probability density of incremental earthquake energies in Southern California during 1981–2011 for *M* ≥ 2 (earthquake catalogue available from the Southern California Earthquake Data Center; http://scedc.caltech.edu). In this case earthquake energies *S* are expressed as *S* = exp(*M*) and the incremental energies as *R* = *S_i_*_+1_ − *S_i_*, where *i* = 1, 2,…, *N*−1 with *N* the total number of earthquakes. Incremental energies are further normalized to zero mean and unitary variance according to $x=\left(R-\langle R\rangle \right)/\sigma_{R}$, where 〈*R*〉 and *σ_R_* are the mean and standard deviation of *R*, respectively. The probability density *p* (*x*) of the normalized incremental earthquake energies deviate from the Gaussian function and instead presents heavy tails and a scaling behavior that can well be described with the *q*-Gaussian distribution for *q* = 1.69 ± 0.08 (Figure 2). This type of scaling enhances the probabilities of large fluctuations that in the case of seismicity designates the occurrence of strong earthquakes immediately after the occurrence of weaker ones. By comparing real earthquake data with the dissipative Olami–Feder–Christensen model (OFC—[49]) in the critical regime [48], interpreted this result as further confirmation for intermittency, self-organized criticality and long-range interactions in the evolution of seismicity.

**Figure 3:**
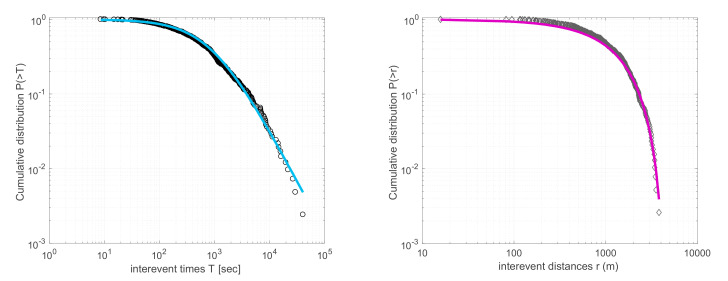
Cumulative distribution functions of the inter-event times (circles) (left) and -distances (diamonds) (right) between the successive earthquakes during the 2008–2009 Yellowstone Lake swarm. The solid lines represent the *q*-exponential distribution (Equation (8)) fitted to the data for the values of *q_T_* = 1.715 and *q_r_* = 0.71. Modified from [38].

Further applications of NESP theory to fracturing and earthquakes concern the spatiotemporal evolution of seismicity from the millimeter scale (laboratory), to tens, hundreds (regional) and thousands of kilometers (global) scale (e.g., [18]). Abe and Suzuki [25,50] showed that the cumulative distribution functions (CDFs) of inter-event distances *P* (>*r*) and inter-event times *P* (>*T*) between successive earthquakes in California and Japan scale according to the *q*-exponential distribution (Equation (8)), for *q*-values of *q_r_* < 1 and *q_T_* > 1, respectively. These results were further tested and verified in acoustic emissions recorded in laboratory experiments [51], in aftershock sequences [52], volcanic seismicity [36,38] and earthquake swarms [53,54], as well as in regional [34,35,37,55,56,57] and global seismicity [40,58]. In Figure 3 we show the CDFs *P* (>*T*) and *P* (>*r*) of inter-event times and distances, respectively, during the 2008–2009 Yellowstone Lake earthquake swarm and the corresponding fits according to the *q*-exponential distribution (after [38]). The *q*-exponential distribution (Equation (8)) describes well the observed distributions for the *q*-values of *q_T_* = 1.715 and *q_r_* = 0.71 (Figure 3). Such results further signify nonlinear dynamics and long-range interactions in the spatiotemporal evolution of seismicity, in agreement with findings from independent methods [59,60,61].

In addition, [9,34] studied the probability density function of inter-event times *T* in nonstationary earthquake timeseries in the Corinth Rift, Southern California and Japan and found a bimodal scaling behavior between two power-law regimes for short and long inter-event times (or waiting times), respectively. This scaling behavior can well be reproduced by a generalized gamma distribution derived within the framework of NESP [62], namely the *q*-generalized gamma distribution that reads as:

(11) $$f\left(T\right)=C{\left(\frac{T}{T_{0}}\right)}^{\gamma -1}\exp_{q}\left(-\frac{T}{T_{0}}\right)$$

where *C* is a normalization constant, *T*_0_ a scaling parameter and *γ* a scaling exponent, while the last term in the right-hand side of the latter equation is the *q*-exponential function (Equation (6)). This type of scaling and the gradual crossover between two power-law regimes indicates clustering effects and correlations at all time scales in the temporal evolution of seismicity, associated with triggered aftershock sequences and long-range interactions in the background activity, respectively [9,10].

### 3.2. Application to Arctic Sea Ice Time Series

The sea ice covering the Arctic ocean is an open, non-equilibrium, multicomponent geophysical system with hierarchic properties [63,64] and well-pronounced scaling behavior [21,65]. Sea ice is a critical parameter for the Earth’s climate system as it insulates the ocean from the atmosphere. As the ice cover deforms and fractures, the albedo decreases allowing the ocean to absorb more shortwaves, so that the ice cover reduces its strength and shrinks during summer, a process that possibly further enhances fracturing [22,66,67]. During winter, on the other hand, sea ice fractures and expands as new ice is produced, a process that customizes the heat and salinity in polar regions [68].

Beyond the key role of sea ice for the Earth’s climate, the sea ice cover further represents a protype for investigating deformation and fracture processes in geophysical systems, as the large lateral extent of the ice cover compared to its thickness allows monitoring of deformation from surface measurements. In addition, monitoring and sampling of deformation in sea ice requires relatively short times, as its deformation develops at much shorter time scales compared to the Earth’s crust.

Previous works have shown that sea ice deformation is accommodated by fracturing processes in a wide range of scales so that strong spatial heterogeneity and intermittency appear in the stress and strain rates, characterized by multifractal scaling properties, extreme fluctuations and long-range temporal correlations [21,69,70]. Various forces drive stresses, strains and fracturing in the sea ice cover [21,71]. Among those, the main component is considered to be the wind that induces stresses and strains with its motion. However, sea ice mechanics and the internal ice stress term seems to be critical in sea ice deformation [72]. In this line, it has been suggested that the intermittence in principal stresses *σ*_1_ and *σ*_2_ and the principal stress direction *θ_s_* does not emerge by the turbulent wind forcing, but it naturally emerges from the fracturing process itself [70].

**Figure 4:**
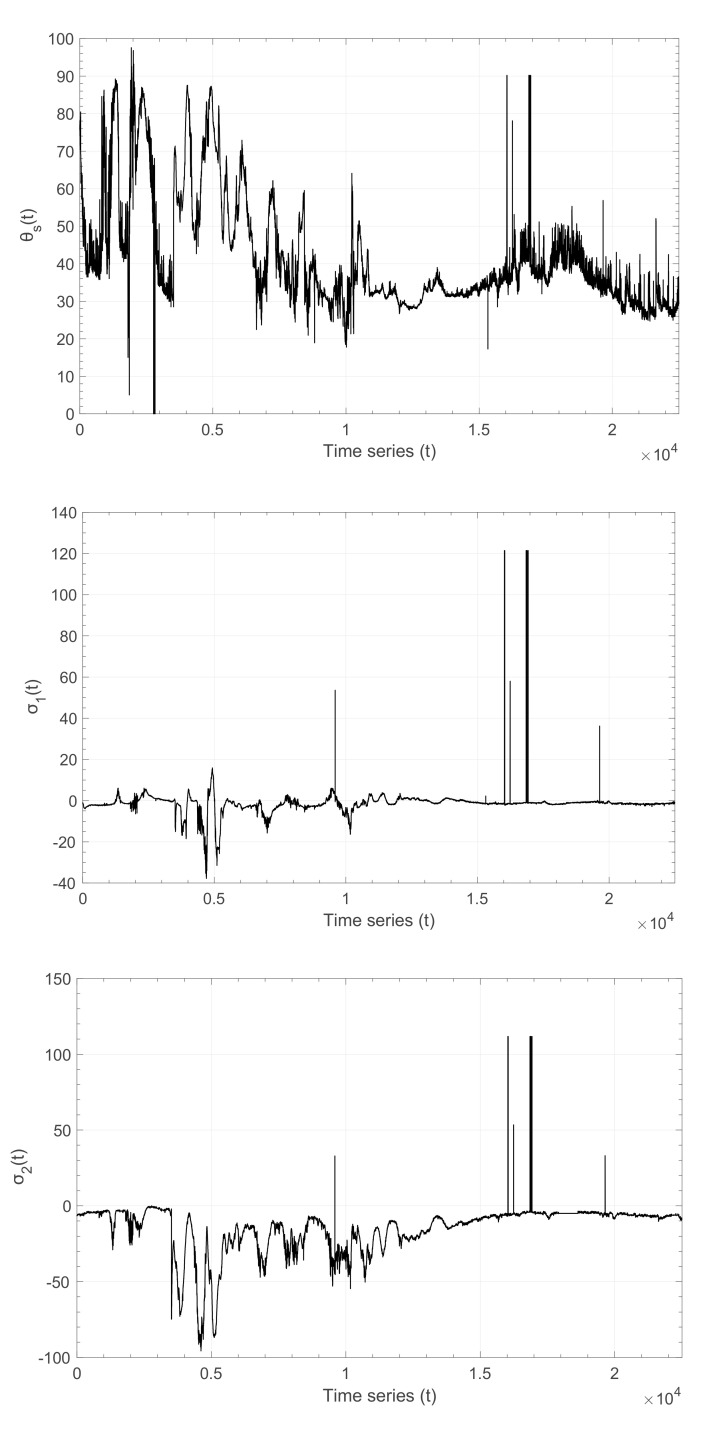
The direction of principal stress *θ_s_* (top) and the principal stresses *σ*_1_ (middle) and *σ*_2_ (bottom) (in kPa) time series, recorded during the CEAREX field campaign within Arctic sea ice.

In the current section we analyze time series of principal stress values *σ*_1_, *σ*_2_ along with the direction of principal stress *θ_s_* within Arctic sea ice recorded during the CEAREX field campaign [73]. In the course of the drift phase of CEAREX during October and November 1988 and at a distance of approximately 230 m from the ship, in-plane compressive stresses were measured in a multi-year ice floe in the eastern Arctic. At this site, ice was in average 1.60 m thick, with thickness variations of less than 20 cm within a 15 m region. Three sensors were installed at roughly the neutral surface of the floe in a rosette pattern to provide calculations of principal stresses *σ*_1_ and *σ*_2_, using a hydraulic fluid-filled flatjack type stress sensor of 20 cm in diameter. The latter provided the estimation of the principal stresses with a resolution of 1.7 kPa. Data sampling, taken once per second, was averaged over two-minutes intervals and stored. The direction of principal stress *θ_s_* and the principal stresses *σ*_1_, *σ*_2_ time series are shown in Figure 4. In the data set analyzed, negative stress values indicate compression and positive stress values tension (Figure 4). The direction *θ_s_* of *σ*_2_ is measured counterclockwise from East. The mean direction *θ_s_* in the data set is 42° with a variance 222, while for the principal stresses *σ*_1_ and *σ*_2_ the mean values are −1.3 kPa and −15.1 kPa and the variances 47.1 and 270.8, respectively.

**Figure 5:**
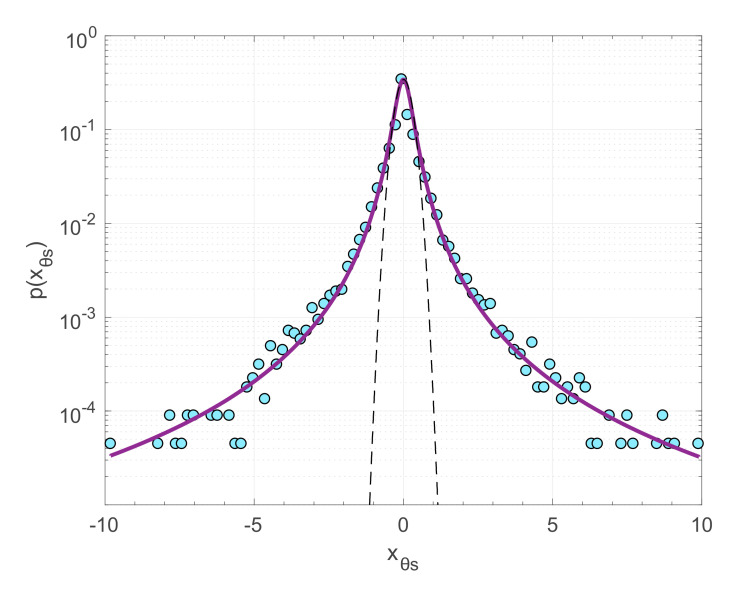

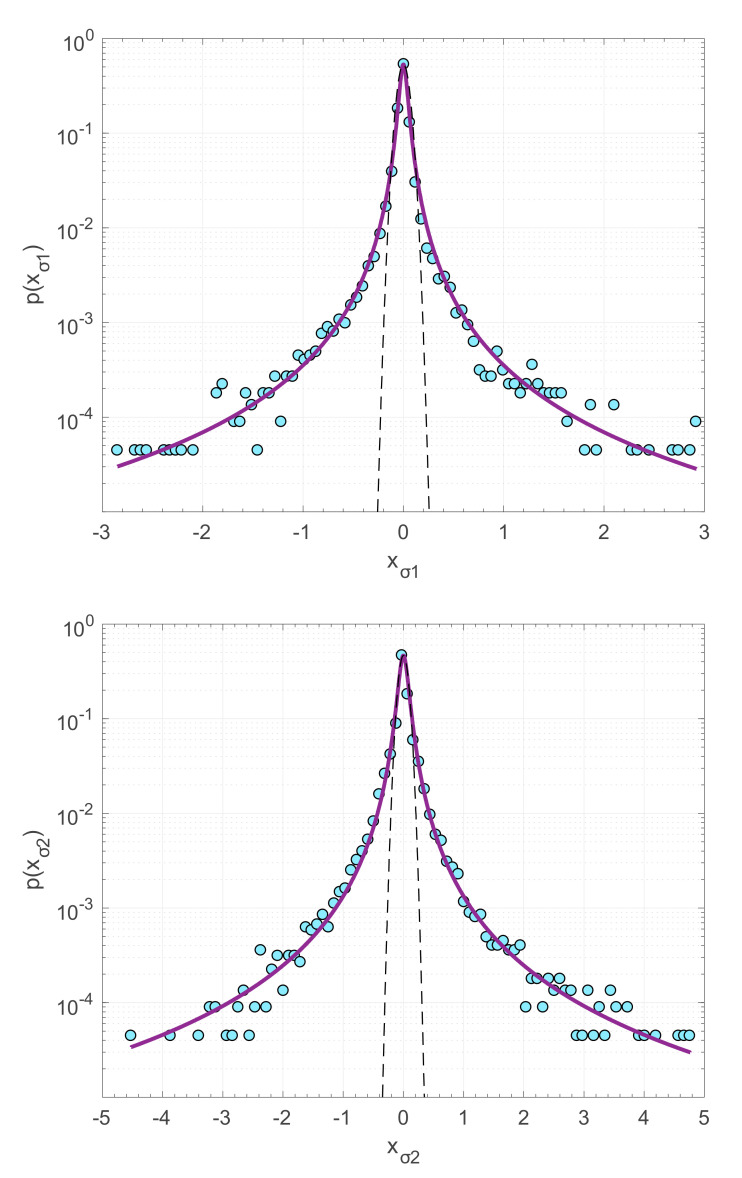
Probability density function of the normalized increments *x* (see text) of *θ_s_*(*t*), *σ*_1_(*t*) and *σ*_2_(*t*) (filled circles) from top to bottom, respectively, the Gaussian fit (dashed black line) and the *q*-Gaussian fit (solid purple line) for the parameter values shown in Table 1.

For the analysis, we consider the increments time series *X*(*t*) of the two principal stress values *σ*_1_ and *σ*_2_ and of the principal stress direction *θ_s_*. The increments time series *X*(*t*) is defined as *X*(*t*) = *S*(*t* + 1) − *S*(*t*), where *S*(*t*) is one of the parameters *σ*_1_, *σ*_2_ and *θ_s_*, respectively. We then construct the probability density function (pdf) *p*(*x*), where $x=\left(X-\langle X\rangle \right)/\sigma_{X}$ with *σ_X_* being the standard deviation of the variable *X*(*t*), normalized to zero mean and unit variance of *X*(*t*). The normalized pdfs *p*(*x*) are shown in Figure 5. From Figure 5 we can immediately verify the departure of the observed pdfs from the classic Gaussian function. Note that in Figure 5 we plot the Gaussian function fitted to the data and not the standard Gaussian function with zero mean and unitary variance. Instead, the observed *p*(*x*) presents heavy tails and scaling behavior that can rather be described with the *q*-Gaussian distribution of the form:

(12) $$f\left(x\right)=A{\left[1-\left(1-q\right)\frac{x^{2}}{B}\right]}^{1/\left(1-q\right)}$$

for the parameter’s values shown in Table 1. The results of this analysis indicate that principal stresses *σ*_1_ and *σ*_2_ and principal stress direction *θ_s_* increments within Arctic sea ice differ from Brownian random noise. Alternately, stress timeseries display long-range time correlations described by the *q*-Gaussian distribution.

## 4. Conclusions

Various aspects of fracturing exhibit complexity. Within this complexity, however, scaling laws seem to apply to the macroscopic properties of fracturing. These laws include the (multi)fractal distribution of fault networks, the G-R scaling relation for the frequency-magnitude distribution of earthquakes and the Omori’s law for the decay rate of aftershocks. While such laws are now well accepted by the scientific community, the fundamental physics in the microscopical level that lead to such patterns remain controversial and to be answered in the future. In the present work we discussed how can the macroscopic properties of fracturing processes and earthquakes be derived by using the first principles of statistical physics. Within this approach, NESP provides a consistent theoretical framework, based on the principle of entropy, for describing some of the essential properties of fracturing, such as (multi)fractality, large fluctuations and long-range correlations that lead to heavy-tailed distributions. Within this framework appropriate probability distributions can be derived that describe some of the collective properties of earthquake and faults, such as fault trace-lengths distributions, the frequency-magnitude distribution of earthquakes, the fluctuations of seismic energy release, or the spatiotemporal scaling properties of seismicity.

Furthermore, we presented for the first time the application of the NESP framework to sea ice stress time series fluctuations. Our results indicate that the principal stresses *σ*_1_ and *σ*_2_ and the principal stress direction *θ_s_* fluctuations within Arctic sea ice exhibits “fat” tails enhanced by long-range correlations, similar to that observed in seismicity. This property further enhances the probability to encounter extreme events that cannot be described by Gaussian statistics. The latter implies that the possibility of experiencing a destructive stress event would be seriously underestimated if dynamic ice stress is assumed to follow a Gaussian distribution. Instead, we have demonstrated that the *q*-Gaussian distribution, derived within the framework of NESP, can adequately describe the scaling behavior of the Arctic sea ice stress fluctuations and the statistics of the extreme events. Hence, the multiscale fracturing processes associated with the deformation and dynamics of Arctic sea ice, characterized by intermittency of strain rates, stress amplitudes and principal stress directions, can be approximated with the *q*-Gaussian distribution. The advantage of considering NESP and the *q*-Gaussian distribution is that, based on the principle of entropy, sea ice mechanics can be associated to statistical physics, while it includes BG statistical physics as a particular case.

Overall, the results presented in a series of recent publications and in the current work support the idea that NESP is an appropriate methodological tool to apply to the macroscopic properties of fracturing processes and earthquakes in terms of probabilities, based on the definition of the relevant microscopic configurations and their interactions. By optimizing the nonadditive entropy *S_q_* using appropriate constraints, a range of power-law to exponential-like distributions are acquired, which are both omnipresent in physical systems. The scaling behavior and the *q*-values of *q* ≥ 1 presented here for sea ice stress timeseries, but also presented elsewhere for the size distribution of fractures, faults and earthquakes for a wide range of scales (e.g., [16,18,19,20,51,74], support the idea that solid Earth materials represent a subadditive complex system with fracturing processes universally characterized by *q*-values of *q* ≥ 1. The compliance of the *q*-exponential or the *q*-Gaussian family of distributions and the macroscopic properties of fracturing implies that the former may act as attractors for fracturing processes. The latter also becomes relevant for a wide class of other complex systems, as diverse as financial markets, living organisms, optical lattices or black holes, among others (e.g., [75,76]), suggesting that fracturing belong to the same universality class as such systems.

Although the results presented here provide a step forward to the better understanding of fracturing phenomena and the underlying physics, the scientific challenge that still remains is to deduce in unified way, using the notions of statistical physics, the physical mechanisms that drive fracture nucleation and evolution. Towards such endeavor, the application of NESP to fracturing phenomena provide a consistent theoretical framework, based on first principles and the concept of entropy, to describe the macroscopic behavior of fracturing processes, where properties such as intermittency, multifractality, long-range correlations and extreme events are intrinsic characteristics of the underlying dynamics.

## Figures and Tables

**Table 1:** Summary table showing the associated values estimated from the analysis for each principal stress (*σ*_1_, *σ*_2_) and stress direction (*θ_s_*), i.e., the *q*-value and *B* from the *q*-Gaussian function (Equation (12)) and their associated uncertainties.

Stress	*q*	*δq*	*B*	*δB*
*σ*_1_	1.85	0.04	0.0017	0.0002
*σ*_2_	1.82	0.12	0.0068	0.0006
*θ_s_*	1.74	0.03	0.078	0.006
